# Structure-function relationships in the sodium chloride cotransporter

**DOI:** 10.3389/fphys.2023.1118706

**Published:** 2023-03-14

**Authors:** Erika Moreno, Diana Pacheco-Alvarez, María Chávez-Canales, Stephanie Elizalde, Karla Leyva-Ríos, Gerardo Gamba

**Affiliations:** ^1^ Department of Nephrology and Mineral Metabolism, Instituto Nacional de Ciencias Médicas y Nutrición Salvador Zubirán, Mexico City, Mexico; ^2^ Escuela de Medicina, Universidad Panamericana, Mexico City, Mexico; ^3^ Unidad de Investigación UNAM-INC, Instituto Nacional de Cardiología Ignacio Chávez and Instituto de Investigaciones Biomédicas, Universidad Nacional Autónoma de México, Mexico City, Mexico; ^4^ Molecular Phisiology Unit, Instituto de Investigaciones Biomédicas, Universidad Nacional Autónoma de México, Mexico City, Mexico

**Keywords:** physiology, sodium transport, structure-function, thiazide, NCC

## Abstract

The thiazide sensitive Na^+^:Cl^−^ cotransporter (NCC) is the principal *via* for salt reabsorption in the apical membrane of the distal convoluted tubule (DCT) in mammals and plays a fundamental role in managing blood pressure. The cotransporter is targeted by thiazide diuretics, a highly prescribed medication that is effective in treating arterial hypertension and edema. NCC was the first member of the electroneutral cation-coupled chloride cotransporter family to be identified at a molecular level. It was cloned from the urinary bladder of the *Pseudopleuronectes americanus* (winter flounder) 30 years ago. The structural topology, kinetic and pharmacology properties of NCC have been extensively studied, determining that the transmembrane domain (TM) coordinates ion and thiazide binding. Functional and mutational studies have discovered residues involved in the phosphorylation and glycosylation of NCC, particularly on the N-terminal domain, as well as the extracellular loop connected to TM7-8 (EL7-8). In the last decade, single-particle cryogenic electron microscopy (cryo-EM) has permitted the visualization of structures at high atomic resolution for six members of the SLC12 family (NCC, NKCC1, KCC1-KCC4). Cryo-EM insights of NCC confirm an inverted conformation of the TM1-5 and TM6-10 regions, a characteristic also found in the amino acid-polyamine-organocation (APC) superfamily, in which TM1 and TM6 clearly coordinate ion binding. The high-resolution structure also displays two glycosylation sites (N-406 and N-426) in EL7-8 that are essential for NCC expression and function. In this review, we briefly describe the studies related to the structure-function relationship of NCC, beginning with the first biochemical/functional studies up to the recent cryo-EM structure obtained, to acquire an overall view enriched with the structural and functional aspects of the cotransporter.

## Introduction

The thiazide sensitive Na^+^:Cl^−^ cotransporter (NCC) is a membrane protein that belongs to the SLC12 family of cation-coupled chloride cotransporters and is localized in the apical membrane of the distal convoluted tubule of the nephron, constituting the main pathway for NaCl reabsorption (Gamba, 2005). In mammalian kidneys, NCC plays a significant role in the homeostasis of the Na^+^, Cl^−^ and K^+^ ions; therefore, when changes in the activity of the cotransporter occurs, arterial blood pressure and K^+^ in plasma changes, resulting in diseases such as Gitelman syndrome (Gitelman et al., 1966; Cruz et al., 2001) and SeSAME syndrome (Bockenhauer et al., 2009; Cuevas et al., 2017), featuring low blood pressure and hypokalemia. Both illnesses produce arterial hypotension accompanied by hypokalemic metabolic alkalosis and hypercalcemia due to poor NCC activity; however, when elevated NCC activity occurs, it leads to the development of a sodium-dependent form of hypertension known as Familial Hyperkalemic Hypertension or Pseudohypoaldosteronism type II, this disease is caused by mutations in the WNK1 and WNK4 kinases (Wilson et al., 2001) or the KLHL3 and Cul3 ubiquitin ligase proteins (Boyden et al., 2012; Louis-Dit-Picard et al., 2012). Therapeutically, NCC is the site of action for thiazide diuretics, and the first line of treatment for arterial hypertension because of their effectiveness and safe use (Whelton et al., 2018). Due to the importance of this cotransporter in the physiology, pathophysiology, and pharmacology of the kidney, the structure-function relationship of this cotransporter has become a topic of interest for many research groups.

## The thiazide sensitive Na-Cl cotransporter (NCC)

**FIGURE 1:**
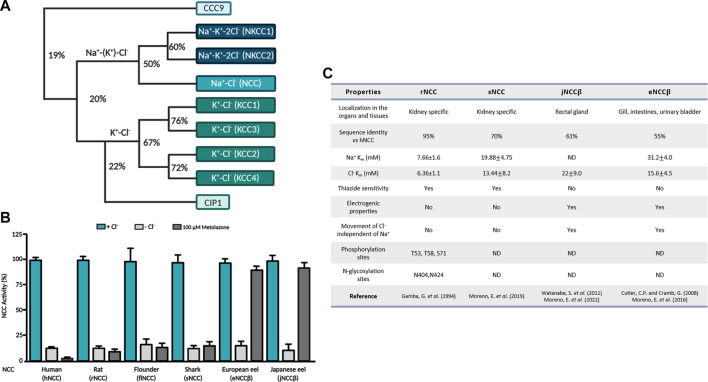
**(A)** Phylogenetic tree of the electroneutral cation-coupled chloride cotransporters of the SLC12 family. Numbers indicate identity percentage. **(B)** Functional expression of different NCCs in *Xenopus laevis* oocytes. NCC activity of different species measured in the absence of extracellular Cl^−^ is represented by the light gray columns, meanwhile in the presence of the thiazide diuretic it is represented by the dark gray columns. **(C)** Summary of the different functional and structural properties of mammalian NCCs vs. NCCs from aquatic species. ND, not defined.

**FIGURE 2:**
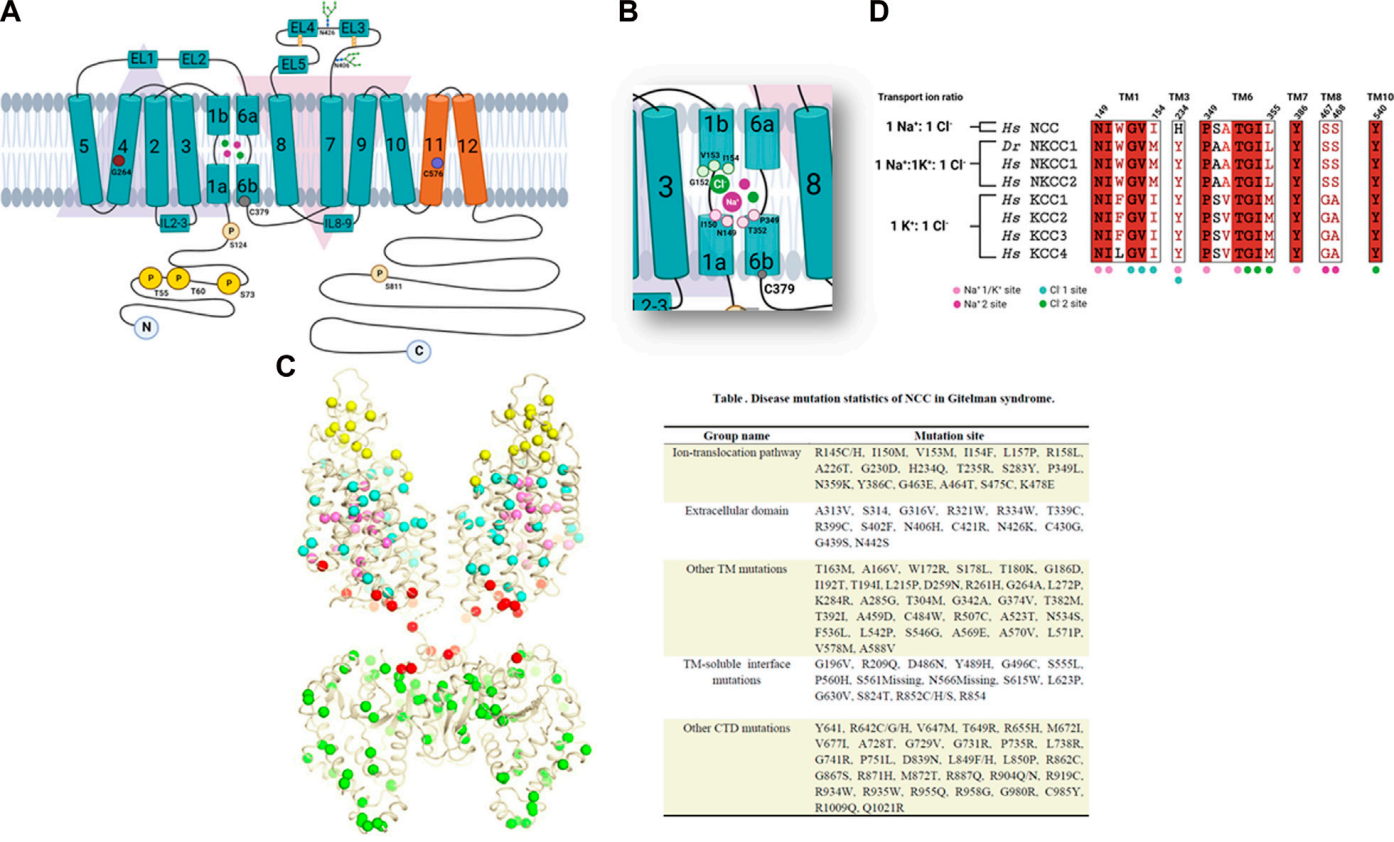
**(A)** Topology map of the NCC. The inverted arrangement of the TM1-5 and TM6-10 domains can be seen in blue and the TM11 and TM12 domains in orange. An extracellular loop with glycosylation sites is observed between the TM7-8 domains. Different amino acids linked to NCC function are also shown. **(B)** Magnified view of the TM1 and TM6 regions with the amino acids involved in ion binding. **(C)** Gitelman syndrome-linked mutations plotted onto the cryo-EM structure of NCC, shown as colored spheres depending on their location. Also grouped by location in the table with their identified mutation site. **(D)** Amino acid sequence alignments analysis of the ion binding sites in human CCCs. Copied from Nan et al. (2022), supplemented with details from studies done by Pacheco-Alvarez et al. (2006), Moreno et al. (2004), Moreno et al. (2006), Moreno et al. (2016), Moreno et al. (2022), Monroy et al. (2000), Castañeda-Bueno et al. (2010), Tutakhel et al. (2018). Created by BioRender.com.

NCC belongs to the SLC12 family of cation-coupled chloride cotransporters (CCC) (Figure 1A, (Gamba, 2005)). Seven members of this family are divided into two main groups, the Na^+^ -dependent cotransporter (NCC, NKCC1, NKCC2) and the Na^+^ independent cotransporters (KCC1, KCC2, KCC3 and KCC4). NKCC1 and NKCC2 translocate Na^+^, K^+^, Cl^−^ unidirectionally with a stoichiometry 1:1:2; meanwhile NCC transports only Na^+^ and Cl^−^, in a 1:1 ratio (Brumback and Staley, 2008; Zhu et al., 2016)*.* The group of K^+^ coupled cotransporters translocate K^+^ and Cl^−^ with a stoichiometry 1:1 (Caron et al., 2000; Kakazu et al., 2000). NCC is expressed in the apical membrane of the distal convoluted tubule of the kidney and in bone osteoblasts (Gamba et al., 1994; Dvorak et al., 2007). NKCC2 is exclusively expressed in the apical membrane of the thick ascending loop of Henle in the kidney (Gamba et al., 1994). Furthermore, NKCC1 is present in many epithelial and non-epithelial cells (Delpire et al., 1994). Inside epithelial cells, it is expressed in the basolateral membrane, except for the choroid plexuses of the brain, where it is expressed in the apical membrane (Delpire et al., 1994; Wu et al., 1998). The degree of identity between these transporters is between 50% and 67% (Figure 1A). The K-Cl branch is composed of four members known as KCC1 through KCC4. KCC2 is expressed in neurons throughout the central nervous system, retina, CA1-CA4 pyramidal cells of the hippocampus, granule cells, cardiomyocytes, glial cells and Purkinje cells (Payne et al., 1996; Hirosawa et al., 1999; Vu et al., 2000; Adragna et al., 2004; Gagnon et al., 2007; Antrobus et al., 2012), while the other 3 KCCs are expressed in various cells throughout the body. The identity between the KCCs is ∼60% and between the Na^+^ dependent and independent branches it decreases to 25% (Gamba, 2005; Arroyo et al., 2013) (Figure 1A). The Na^+^ coupled cotransporters transfer ions from the extracellular space to the cytoplasm; meanwhile, the KCCs mediate the efflux of ions from the cells (Gamba, 2005). Lastly, two additional members of the family that were identified were CIP1 and CCC9; however, their function and substrate identity are not well defined (Gamba, 2005). At first, it was thought that these two cotransporters played a role in Na^+^ and K^+^ coupled Cl^−^ transport; however, a decrease in NKCC1 and NKCC2 function was observed when they were injected along with CIP1 in HEK-293 cells and *X. laevis* oocytes (Caron et al., 2000). Subsequently, through biochemical analyses, CIP1 was capable of activating KCC2 through heteromer formation (Wenz et al., 2009). On the other hand, CCC9 has been associated with both amino acid and polyamine transport (Daigle et al., 2009). Grozio *et al.*, (Grozio et al., 2019), recently proposed that CCC9 is a nicotinamide mononucleotide (NMN) transporter that is regulated by NAD+ (Nicotinamide adenine dinucleotide). Analysis made by Schmidt and Brenner (2019), however, demonstrated the lack of methodological evidence that is needed to consider CIP1 as an NMN transporter, therefore, CCC9 should be considered a Na^+^ transporter instead. The first member of the CCC family identified at the molecular level was NCC. Gamba *et al.*, identified the cDNA from the urinary bladder of the winter flounder (flNCC) and later on from rat kidney (Gamba et al., 1994) (rNCC). NCC has been cloned from several species, including the hNCC (Mastroianni et al., 1996; Simon et al., 1996), mouse (mNCC) (Kunchaparty et al., 1999), rabbit (rbNCC) (Velázquez et al., 1998), zebrafish (zfNCC) (Wang et al., 2009), shark (sNCC) (Moreno et al., 2019), Japanese and European eels (jNCC and eNCC) (Cutler and Cramb, 2008; Watanabe et al., 2011). The mammalian NCC is a110 kDa membrane protein with 1,002 amino acid residues, and is glycosylated (Bostanjoglo et al., 1998). The NCC structure is composed of two grand cytosolic domains according to hydropathic analyses (Kyte and Doolittle, 1982), that correspond to the intracellular N-terminal and C-terminal ends that are connected by 12 central transmembrane helices (TM), (Figure 2A). The protein is glycosylated in the asparagine residues (N406 and N426), located in the extracellular loop that binds TM7 and TM8 (EL4) (Hoover et al., 2003; Gamba, 2005; Nan et al., 2022).

## Functional properties of NCC

The cloning of NCC opened the possibility of exploring the distinct structural and functional aspects of the protein, Monroy et al. (2000), Vázquez et al. (2002) studied the kinetic properties of flNCC and rNCC, revealing that rNCC has a higher affinity for both transported ions, with a Michaelis-Menten constant (*K*_*m*_) of 7.66 ± 1.6 mM for Na^+^ and 6.36 ± 1.1 for Cl^−^, compared to flNCC that displayed *K*_*m*_ values of 25.06 ± 0.4 for Na^+^ and 13.56 ± 0.2 mM for Cl^−^ (Figure 1C). The Hill coefficient of sodium and chloride in both cotransporters was close to 1, demonstrating an electroneutral cotransporter. NCC is specifically inhibited by thiazide-type diuretics, with a higher affinity in the rNCC orthologue than in the flNCC (Figure 1C). Throughout the last few years, the kinetic properties of sNCC, eNCC and jNCC have also been studied, as shown in Figures 1A, C a lower affinity for transported ions is found in these aquatic species (Moreno et al., 2016; Moreno et al., 2019; Moreno et al., 2022). As we previously described, different NCC species demonstrate different *K*_*m*_ values of the ions transported. Currently, it is known that NCC’s ion binding sites are highly conserved in other members of the SLC12 family (Nan et al., 2022); however, it is difficult to comprehend the conformational changes that occur in NCCs of different species due to the differing percentages of identity in their amino acid sequences. At this moment, the role of residues near the ion binding sites remains unclear; nevertheless, they are certainly responsible for determining the different affinities for ion transport. NCC expression has been limited to the distal convoluted tubule of different species. However, it has been reported that in the European and Japanese eels, two isoforms of NCC exist and originate from different genes. Cutler and Cramb (2008) cloned NCC alpha (eNCC⍺) from renal tissue and NCC beta (eNCCβ) from intestinal tissue of the European eel; the identity with mammalian NCC for eNCC⍺ and eNCCβ is ∼70% and 55% respectively. Moreno et al. (2016) observed that although eNCCβ is a Na-Cl cotransporter, it is nevertheless insensitive to thiazides (Figure 1B), because concentrations of up to 10^–3^ M of different thiazides could not inhibit their function. Watanabe et al. (2011) reported the cloning of NCCβ derived from intestinal tissue of a Japanese eel (jNCCβ), observing that it was sensitive to hydrochlorothiazide in prepared rectal sacs. eNCCβ and jNCCβ were 98% identical, only differing by 13 amino acids (Moreno et al., 2016). By utilizing *Xenopus laevis* oocytes as a functional expression system, Moreno *et al.,* recently reported that jNCCβ is also not sensitive to thiazides, highlighting the different findings in the cotransporter’s diuretic sensitivity (Figure1 B) (Moreno et al., 2022).

NCC has been described as the electroneutral cotransporter incapable of producing an electrical current. However, the functional characterization of eNCCβ and jNCCβ demonstrated an electrical current dependent on the voltage (Moreno et al., 2022) of *X. laevis* oocytes. Studies found that although most Na^+^ and Cl^−^ transport was interdependent on the Hill coefficient of 1:1, the uptake of ^22^Na^+^ induced by NCCβ depended utterly on the presence of chloride; meanwhile the uptake of ^36^Cl^−^ did not depend entirely on Na^+^; therefore, suggesting the generation of a remainder Cl^−^ current. To verify this**,** the current was inverted to 20 mV of which the elimination of Na^+^ of the washed solution was unaffected but was eliminated completely when Cl^−^ was absent, strongly suggesting that it was a chloride-dependent current. Thus, the non-renal NCCβ variant of NCC is an electrogenic Na-Cl cotransporter that is not sensitive to thiazides; the characterization of the generated current by European and Japanese eel NCCβ isoforms will be the subject of many future studies.

## Amino acids linked to NCC function

### Transmembrane regions (TM)

Since the cloning of NCC in different species, the primary sequences of the cotransporter were of great use in initializing comparative structural analyses. The identity of hNCC is ∼90% with other mammalian NCCs and ∼60% with flNCC, meanwhile the mammalian NCC exhibits ∼55% identity to that of the eel’s NCCβ (Figure 1C). The structural comparisons determined that the TM segments are where the major percentage of identity is found (∼80%). In contrast, the N-terminal and C-terminal regions are less similar, sharing around 20% and 55%. In 2003, Hoover *et al.* (Hoover et al., 2003) demonstrated that rNCC has two N-glycosylation sites located in EL4. N404 and N424 are essential for proper rNCC expression and function. Mutated clones in any of the two sites decreased rNCC function and when both sites were absent more than 80% of its function was reduced. On the contrary, the elimination of N-glycosylation resulted in an exaggerated increase in rNCC’s sensitivity to metolazone. Moreno et al. (2006) performed an alignment of NCC’s EL4 of different species, and identified the already defined glycosylation sites in mammals and discovered three additional sites in flNCC, N403, N414 and N432. The first site N403 is the equivalent to N404 in rNCC and is conserved in all the species studied. N414 is only found in flNCC meanwhile N432 was identified in both flNCC and zfNCC sequences. Functional analyses of mutated and cloned N-glycosylation sites in flNCC did not display any disturbance in the cotransporter’s function nor in its metolazone sensitivity. Only a partial reduction in function was observed in the clone that lacked all three sites. These studies concluded that the N-glycosylation sites did not appear to be determinants of NCC’s functional properties.

By constructing chimeric proteins between mammalian NCC and flNCC, Moreno et al. (2006) determined that Cl^−^ transport in NCC is coordinated by the TM1-7 region, meanwhile thiazide binding is coordinated by the TM8-12 region. Previously, the importance of TM4 in the Cl^−^ affinity to NCC has been reported (Moreno et al., 2004). In a study of a single nucleotide polymorphism (SNP), glycine-264 (NCC-G264) was in this segment, revealing that NCC’s affinity to Cl^−^ significantly increases at least one order of magnitude compared to that of a native transporter (Figure 2A) (Moreno et al., 2004). This polymorphism was later shown to affect the response to loop diuretics since the activity of NCC in DCT compensates part of the natriuresis induced by the inhibition of salt reabsorption in the TAL (Vormfelde et al., 2007). Furthermore, the importance of TM8-12 in thiazide affinity was highlighted in a study by Castañeda-Bueno et al. (2010). They found that TM11, specifically cysteine-575 in rNCC (substituted by a serine in flNCC) handles the difference in diuretic affinity in rNCC and flNCC.

Although the above studies brought us closer to identifying structure-function aspects of NCC, for many years the key amino acids involved in ion transport or thiazide remained unidentified. However, recent high-resolution studies on other members of the CCC family have found that ion and diuretic binding to the CCCs involves complex processes in different TM regions (Reid et al., 2020; Chew et al., 2021; Zhang et al., 2021).

### N-terminal and C-terminal domains

As shown in other studies, the TM domain of NCC has been an object of study to understand ion transport and inhibition by thiazides. It was revealed that both the N-terminal and C-terminal domains do not participate in these functions (Tovar-Palacio et al., 2004; Moreno et al., 2006); however, specific amino acids involved in other NCC functions have been found in these domains.

It is known that the members of the SLC12 family are regulated by a cascade of kinases, in which With-No-Lysine kinase (WNK) phosphorylate and regulate the STE20/SPS1related proline-alanine-rich protein kinase (SPAK) and the Oxidative Stress-Responsive kinase 1 (OSR1). SPAK and OSR1 also phosphorylate CCCs (Richardson et al., 2008; Gagnon and Delpire, 2012; Alessi et al., 2014). Pacheco-Alvarez et al., demonstrated that the phosphorylation of rNCC occurred in residues: threonine-53 (T53), threonine-58 (T58) and serine-71 (S71) (Pacheco-Alvarez et al., 2006) (Figure 1C), this was later biochemically demonstrated by Richardson et al. (2008). In 2012, two phosphorylation sites were reported, Rosenbaek et al. (2012) identified a phosphorylation site at the N-terminal end of rNCC, serine-124 (S124). The phosphorylation of this residue was associated with the treatment of arginine vasopressin or a low sodium dietary state but was not related to with the participation of SPAK/OSR1. Tutakhel et al. (2018) studied a phosphorylation site exclusively found in the C-terminal domain of hNCC, serine-811 (S811), whose phosphorylation was necessary for the phosphorylation of T55 and T60 (Figure 2A). Lastly, many amino acid residues in NCC have been studied because of their participation in Gitelman syndrome (GS) [OMIM 263800], defined as an autosomal recessive salt-wasting tubular disease caused by mutations in NCC (Gitelman et al., 1966; Cruz et al., 2001).

Studying these mutations has led to our understanding that the disruption of the protein’s function can be because of alterations in the protein’s synthesis, processing, membrane insertion, and degradation (Sabath et al., 2004). By searching the human genome database (HGMD, professional 2018.3), over 500 different mutations along NCC have been linked to Gitelman’s disease. Nan et al. (2022) recently mapped out the numerous Gitelman syndrome-related mutations onto their NCC cryo-EM (single-particle Cryogenic Electron Microscopy) structure and classified them in five groups according to their function or location. In Figure 2C, the mutations appear as various colored spheres: located extracellularly (yellow); in the ion translocation pathway (pink); other TM domains (cyan); TM-C-terminal interface (red); and in the N-terminal domain (green).

## SLC12 family structures in high resolution

### Amino acid residues involved in ion binding

The crystallographic structure of the C-terminal domain of a prokaryote CCC (archaea *Methanosarcina acetivorans*), was the first approach to reveal the architecture of this family. By using X-rays, Warmuth et al. (2009) demonstrated for the first time that the C-terminal domain was composed of organized ⍺/β sheets in two subdomains that were structurally related and appeared similar to universal stress proteins, USP (Kvint et al., 2003). It was also discovered that this domain was capable of forming dimers through a hydrophilic interface rich in arginine and glutamate residues (Warmuth et al., 2009). Since 2013, most of these atomic structures deposited in protein banks (PDB) were determined by X-ray crystallography; however, the method was limited to many mammalian membrane proteins. In the last decade, cryo-EM became an effective and reliable technique for achieving an atomic resolution of protein structures. In 2019, Chew et al. (2019) reported the first high resolution structure of a member of the CCC family, NKCC1 from the *Danio rerio* zebrafish (zfNKCC1).

The images demonstrated that the conformation of the TM domains of zfNKCC1 is like that of the Amino Acid-Polyamine-Organocation (APC) family with inverted architecture of the TM1-5 and TM6-10 domains. Two extracellular loops were found in between TM7-8 (EL4) and between TM5-6 (EL3) thus forming a cap domain. The cap domain is necessary for the expression and regulation of transport of human NKCC1 (hNKCC1) (Ye et al., 2012). The zfNKCC1 structure was captured in a partially open inward conformation, forming a vestibule comprising of the TM1, TM3, TM6, and TM8 regions that extends to the intracellularly surface; different electrostatic charges exist within the vestibule, which facilitates ion accommodation. Solid evidence suggests that K^+^ binding is coordinated by a hydroxyl group of tyrosine-305 (Y305), which is conserved in all K^+^ binding cotransporters (NKCC and KCC) but not in the K^+^ -independent NCC (which contains histidine instead of tyrosine). Besides Y305, asparagine-220 (N220), isoleucine-221 (I221), proline-417 (P417) and threonine-420 (T420) are involved in K^+^ binding in the TM1 and TM6 regions. In the case of the Na^+^ binding site, amino acids found in TM1 (leucine-219 (L219), tryptophan-222 (W222), and in TM8 (serine-538 years serine-539 (S538 and S539) are involved. This site is highly conserved in the transporters that pertain to the APC family, this is supported by the closeness in an overlap of the zfNKCC1 structure onto the Na^+^ bound sialic acid transporter of the *P. mirabilis* (SiaT) structure. In the case of Cl^−^, dynamic simulations involving all the atoms (Na^+^, K^+^, and Cl^−^) revealed three binding sites for this ion. Nonetheless, it was proposed that Cl^−^ bonded to only two sites, the first (site 1) was coordinated by K^+^ and the residues: valine-224 (V224) and methionine-225 (M225). The second (site 2) is coordinated by the residues: glycine-421 (G421), leucine-423 (L423) and tyrosine-611 (Y611). Since the cryo-EM structure of the hNKCC1 was similar to that of zfNKCC1, the ion binding sites involved the same residues (Zhang et al., 2021). Furthermore, the general architecture of KCCs is also similar to that of zfNKCC1 and hNKCC1 (Zhang et al., 2021). In the crystallographic structure of KCC, the TM regions share the same disposition observed in the APC family, and the overlap of both KCC and APC structures presents conserved ion binding sites (Reid et al., 2020; Xie et al., 2020). However, when compared to the Na^+^-dependent cotransporters, a unique aspect pertaining to KCCs is a longer extracellular loop in between TM5-6 (Liu et al., 2019).

Recently, a high resolution cryo-EM structure of NCC was achieved (Nan et al., 2022). The map of the TM regions permitted the construction of precise atomic models of this region and EL4 as well. Unfortunately, a moderately clear C-terminal domain was obtained. The NCC structure displayed a dimeric architecture and the TM regions adopted a similar conformation observed in members of the APC family (Figure 2A). Notably, TM1 and TM6 helices were interrupted in the medial region, forming discontinuous sites adequate for ion binding. Two extracellular loops were observed superior to the TM domain, one loop connected to TM5 and TM6, and another to TM7 and TM8. In the first loop, two helices are formed (EL1 and EL2); meanwhile in the second loop, there are three helices (EL3 to EL5). Two glycosylation sites were also observed (N406 and N426) that are essential for efficient NCC expression and activity (Hoover et al., 2003). Nan *et al.* defined two Na^+^ binding sites and two Cl^−^ binding sites in their NCC structure. Both Na^+^1 and Cl^−^1 binding sites are located in a pocket formed by the antiparallel broken helices of TM1 and TM6, and correspond to the conserved K^+^ and Cl^−^1 binding sites in NKCC1 and KCCs (Chew et al., 2019; Liu et al., 2019; Xie et al., 2020; Neumann et al., 2022) (Figures 2A, B, D).

The Cl^−^1 binding site is coordinated by the main-chain amide group of glycine-152 (G152), valine-153 (V153), and isoleucine-154 (I154 of TM1) and the hydroxyl group of serine-350 (S350), that also weakly coordinates this ion. The Na^+^1 binding site is accommodated by the main-chain carbonyl groups of asparagine-149 (N149), isoleucine-150 (I150), proline-349 (P349), and threonine-352 (T352) (Figure 2B), Nan et al. (2022). Notably, mutations in I150M, V153M, I154F y P349L, can cause Gitelman syndrome, demonstrating the important role of these residues in NCC transport activity (Figure 2C). The histidine (H234) residue is located in the TM3 region, near Na^+^1 and Cl^−^1 and is considered to be a key residue in Na + transport in NCC since other CCCs contain a tyrosine residue instead. Another conserved residue, tyrosine-386 (Y386) in TM7 interacts with the main chain carbonyl groups of Ile-150 and Ser-350. Y386 is highly conserved in all the members of the SLC12 family and can be essential to ion coordination and coupling involving TM1, TM6, and TM7 (Figure 2D).

The second Cl^−^ site (Cl^−^2) is also conserved in both sodium as well as potassium coupled cotransporters (Delpire and Guo, 2020; Chew et al., 2021; Portioli et al., 2021). Additionally, the second Na^+^ binding site (Na^+^2) identified in NCC was also reported in NKCC1 (Chew et al., 2019; Neumann et al., 2022; Zhao et al., 2022) (Figure 2D). Na^+^2 is coordinated by the hydroxyl groups of serine-467 (S467) and serine-468 (S468) of TM8 as well as alanine-464 (A464), tryptophan-151 (W151) and leucine-148 (L148). Sequence alignments demonstrated that the two serine residues that coordinate Na^+^2 are also conserved in Na^+^ dependent CCCs but are absent in Na^+^ independent cotransporters (Nan et al., 2022).

Finally, the cryo-EM structure of NCC reported by Nan *et al.*, provides an improved and detailed view of the cotransporter and its defined ion bindings sites, paving the way in determining ion transport and developing NCC specific medication in the near future.

## Concluding comments

The advances in the structural elucidation of the CCC family members through the use of the cryo-EM technique along with a functional characterization of the different cotransporters have brought out substantial information about NCC function at a molecular level. Although there are still many questions left unanswered, these impressive studies provide a solid foundation to understand the structure-function relationship of the cotransporter. Hopefully, soon, the creation of a high-resolution structure will allow us to visualize the interactions between NCC and thiazides, which will shed light on the molecular basis of their pharmacology and will lead to the development of medication with better therapeutic benefits and use.
